# Beta-Thalassemia Intermedia: A Single Thalassemia Center Experience from Northeastern Iraq

**DOI:** 10.1155/2020/2807120

**Published:** 2020-02-28

**Authors:** Shaema Salih Amin, Sana Dlawar Jalal, Kosar Muhammed Ali, Ali Ibrahim Mohammed, Luqman Khalid Rasool, Tara Jamel Osman

**Affiliations:** ^1^Sulaymaniyah Directorate of Health, Hewa Oncology Hospital, Sulaymaniyah, Iraq; ^2^Department of Pathology, College of Medicine, University of Sulaymaniyah, Sulaymaniyah, Iraq; ^3^Department of Medicine, College of Medicine, University of Sulaymaniyah, Sulaymaniyah, Iraq; ^4^Thalassemia and Congenital Blood Disorders Center, Sulaymaniyah 46001, Iraq

## Abstract

**Objective:**

To determine the molecular characterization and disease-associated complications of beta-thalassemia intermedia (*β*-TI) patients in Sulaymaniyah province, northeastern Iraq.

**Methods:**

A total of 159 *β*-TI) patients in Sulaymaniyah province, northeastern Iraq. *β*-TI) patients in Sulaymaniyah province, northeastern Iraq.

**Results:**

Nineteen different *β*-globin gene mutations arranged in 37 various genotypes were determined. The most frequent were IVS-II-I (G>A) (47.2%), followed by IVS-I-6 (T>C) (23.3%) and IVS-I-110 (G>A) (5%). Among disease-related morbidities documented, bone disease amounted to 53% (facial deformity and osteoporosis), followed by endocrinopathies 17.6% (growth retardation and subclinical hypothyroidism), cholelithiasis 13.8%, pulmonary hypertension 11.3%, and abnormal liver function test 7.5%, whereas venous thrombosis, extramedullary hemopoiesis, and leg ulcer were less frequently observed. Age ≥ 35 and female sex were risk factors for cholelithiasis, while age was an independent risk for hypothyroidism and female sex was associated with increased risk for osteoporosis. Mean serum ferritin of ≥1000 *μ*g/L was associated with an increased risk of osteoporosis, whereas chelation therapy was protective for a multitude of other complications. Transfusion, on the other hand, increased the risk of osteoporosis, yet it was protective for cholelithiasis and hypothyroidism. Moreover, splenectomy was protective for cholelithiasis, although it was an independent risk for hypothyroidism. Finally, hydroxyurea was associated with an increased risk of osteoporosis, while it was protective for cholelithiasis. *Discussion and Conclusion. β*^+^-thalassemia mutation had contributed to 41.25 of families with a less severe *β*-thalassemia phenotype in the northeastern part of Iraq, justifying the need to investigate the contribution of genetic modifiers in ameliorating disease severity. In addition, the substantial number of *β*-TI patients developed disease-related morbidities, which necessitates the need for more appropriate clinical management with earlier intervention.*β*-TI) patients in Sulaymaniyah province, northeastern Iraq. *μ*g/L was associated with an increased risk of osteoporosis, whereas chelation therapy was protective for a multitude of other complications. Transfusion, on the other hand, increased the risk of osteoporosis, yet it was protective for cholelithiasis and hypothyroidism. Moreover, splenectomy was protective for cholelithiasis, although it was an independent risk for hypothyroidism. Finally, hydroxyurea was associated with an increased risk of osteoporosis, while it was protective for cholelithiasis. *Discussion and Conclusion. β*^+^-thalassemia mutation had contributed to 41.25 of families with a less severe *β*-thalassemia phenotype in the northeastern part of Iraq, justifying the need to investigate the contribution of genetic modifiers in ameliorating disease severity. In addition, the substantial number of *β*-TI patients developed disease-related morbidities, which necessitates the need for more appropriate clinical management with earlier intervention.*Discussion and Conclusion. β*^+^-thalassemia mutation had contributed to 41.25 of families with a less severe *β*-thalassemia phenotype in the northeastern part of Iraq, justifying the need to investigate the contribution of genetic modifiers in ameliorating disease severity. In addition, the substantial number of *β*-TI patients developed disease-related morbidities, which necessitates the need for more appropriate clinical management with earlier intervention.*β*-TI) patients in Sulaymaniyah province, northeastern Iraq. *β*-TI) patients in Sulaymaniyah province, northeastern Iraq.

## 1. Introduction

Beta-thalassemia (*β*-thal) is one of the most widely distributed autosomal recessive disorders which affects the *β*-globin gene of the hemoglobin with considerable predominance in the thalassemia belt area, including, Iraq [1]. The disease is characterized by diminished (*β*^+^, *β*^++^) or lack (*β*^0^) of generation of the *β*-globin chain [2].

All over the world, over 350 various mutations of the *β*-globin gene were recorded [3, 4]. The genetic pathophysiology of TI results from marked molecular heterogeneity of the *β*-globin gene with the inheritance of one or two mutations interacting with many genetic variables among which is concomitant *α*-thalassemia and polymorphism at the three major quantitate loci (QTL) to increase Hb F production [5].

Numerous serious long-term sequelae have been determined in *β*-TI related to the degree of anemia and iron deposition [6]. The severity of anemia stems from ineffective erythropoiesis and peripheral hemolysis of the mature red blood cells. The morbidities include facial deformity, pulmonary hypertension (PHT), thromboembolic complication, extramedullary hemopoiesis (EMH), and endocrine complications (hypothyroidism, diabetes mellitus, hypogonadism, and osteoporosis) [7, 8].

*β*-Thal is a major public health burden in the Kurdistan Region of Iraq with an estimated carrier frequency in Sulaymaniyah of 4.1% [9]. The current study had investigated the molecular characterization of *β*-TI in the northeastern part of Iraq and attempted to evaluate the disease-associated complications in this subgroup of thalassemia.

## 2. Patients and Methods

### 2.1. Patients

This study was conducted between July 2018 and August 2019 enrolling a total of 159 *β*-thalassemia intermedia patients from 114 registered families at the Sulaymaniyah Thalassemia Care Center through random selection from the targeted population to avoid bias. All medical files of these patients were reevaluated. The diagnostic criteria were based on (i) age at diagnosis or initiation of transfusion ≥ 2 years, (ii) maintain hemoglobin ≥ 7 − 7.5 g/dL, and (iii) intermittent transfusion requirements [10, 11].

The study was approved by the ethical committee at the College of Medicine, University of Sulaymaniyah, Iraq, and informed consent was obtained from all enrollees.

### 2.2. Clinical Assessment

A standardized comprehensive questionnaire was used to obtain detailed clinical information regarding demographic data, age at the initiation of transfusion and diagnosis, time interval of blood transfusion in the past year, family history of thalassemia, rate of consanguinity, and parenthood. All patients were physically examined regarding skeletal facial deformity, height (to detect growth retardation when height > 2SD below 3rd percentile for the mean age and gender), and recurrent leg ulceration. The sizes of the spleen (unless splenectomized) and liver were also determined, and gall bladder stones were screened by ultrasound. Details regarding pathological fractures, diabetes, documented venous thrombosis, and extramedullary hemopoiesis, with medications used for iron chelation and hydroxyurea were obtained from patients' files.

### 2.3. Laboratory Investigations

#### 2.3.1. Hematological Tests

Five milliliters of venous blood sample was aspirated and transferred equally into an EDTA anticoagulated tube and a plain tube. The anticoagulated blood was used to perform red cell parameters using a fully automated hematology analyzer (Swelab, Spånga, Sweden), and Hb A_2_ and Hb F quantification was performed using high-performance liquid chromatography (HPLC) (D-10, Bio-Rad Laboratories, Hercules, CA, USA). These investigations were performed just before receiving the next blood transfusion, while for patients who were on regular transfusion sessions (every 3-4 months), the HPLC results were recorded at the time of first presentation or when diagnosis was documented. The remaining EDTA blood sample was centrifuged, and a buffy coat was separated and frozen at −20°C for later DNA extraction.

#### 2.3.2. Biochemical and Other Tests

The serum was utilized for the estimation of ferritin by an ELISA method (Biokit, USA), as well as serum glucose (mg/dL) and serum alanine transaminase (ALT) (U/L). Virological screening included Hepatitis B surface antigen (HBsAg) (Plasmatec Laboratory Products, UK), antihepatitis C virus (HCV) antibodies (Plasmatec Laboratory Products, UK), and Human Immune Deficiency Virus (HIV) antibodies 1 and 2 (Plasmatec Laboratory Products, UK). Thyroid function tests including thyroid-stimulating hormone (TSH) and free T4 were estimated using enzyme immunoassay (TOSOH, Japan). Thyroid-stimulating hormone and free T4 were measured in all TI patients ≥10 years annually [12]. Patients with elevated TSH > 4.7 *μ*IU/mL and low free T4 < 0.8 ng/dL were labeled as overt hypothyroidism, while subclinical hypothyroidism was diagnosed if patients had elevated high TSH > 4.7 *μ*IU/mL in conjunction with normal free T4 > 0.8 ng/dL [13].

#### 2.3.3. Molecular Tests

The genomic DNA was isolated from peripheral blood leukocytes which were collected in a 0.5 M K_2_EDTA anticoagulated tube based on the phenol-chloroform procedure [14]. The DNA samples were then amplified in a multiplex polymerase chain reaction (PCR), followed by reverse hybridization to specific oligonucleotide arrays fixed on test strips which are constructed to determine a panel of 22 relatively common Mediterranean *β*-thal mutations using the *β*-globin StripAssay MED® Kit (ViennaLab Diagnostics GmbH, Vienna, Austria). All steps were performed as recommended by the manufacturer's instructions. The mutations include -101 (C>T); -87 (C>G); -30 (T>A); codon 5 (-CT); codon 6 (G>A); codon 6 (A>T); codon 6 (-A); codon 8 (-AA); codon 8/9 (+G); codon 15 (G>A); codon 27 (G>T); IVS I-1 (G>A); IVS I-5 (G>C); IVS I-6 (T>C); IVS I-110 (G>A); IVS I-116 (T>G), IVS I-130 (G>C); codon 39 (C>T); codon 44 (-C); IVS II-1 (G>A); IVS II-745 (C>G); and IVS II-848 (C>A). In 17 cases, when no or one mutation was revealed by reverse hybridization, both EDTA blood and DNA samples were sent to Kariminejad-Najmabadi Pathology and Genetic Center in Tehran, Iran, for direct sequencing of the whole *β*-globin gene (*HBB*) using the ABI Prism Big Dye Terminator Cycle Sequencing Ready Reaction kit and the ABI Prism 377 DNA Automatic Sequencer (Perkin Elmer, Foster City, CA). Forward primer A (CTTAGGCTGCTGGTGGTCTACC) and reverse primers B (AGCACTTTCTTGCCATGAGCC) and C (ATGCACTGACCTCCCACATTCC) corresponded to positions 391, 469, and 1740, respectively. We screened for -*α*^3.7^, -*α*^4.2^, and the -^Med-1^*α*-thal deletions as well as *α*-gene triplication by gap PCR [15].

### 2.4. Echocardiography

All patients at their regular visiting dates were subjected to periodic echocardiographic evaluation. Those who had a pulmonary artery systolic pressure (PASP) ≥ 25 mmHg combined with exertional dyspnea at rest without evidence of left-side heart failure were regarded as pulmonary hypertension [16].

### 2.5. Bone Mineral Density Evaluation

Dual Energy X-ray Absorptiometry (DEXA) scan was performed annually for patients with ages of ≥10 years [12]. The diagnosis of osteoporosis was based on finding a decreased bone mass density with a *T* − score≤−2.5SD based on BMD measurement at the lumbar spine and hip [17, 18].

### 2.6. Statistical Methods

SPSS version 25.0 (Armonk, NY; IBM Corp., USA) for Windows was used for data analysis. Means and SD were calculated in continuous data and the frequency proportion for categorical data. ANOVA test for the statistical differences in means and chi-square for the relation of different genotype groups, complications, and other categories to different parameters was calculated. Multivariate logistic regression analysis was done for each complication as a dependent variable to determine the independent effect of study parameters. The *P* value was considered statistically significant at a level of <0.05.

## 3. Results

### 3.1. Patient Characteristics

One hundred fifty-nine *β*-thalassemia intermedia (*β*-TI) patients were enrolled, including 90 (56.6%) males and 69 (43.4%) females, with a male : female ratio of 1.3 : 1. The patients' ages ranged between 1.4 and 54 years with a median of 15 yrs. Age at diagnosis varied between 1 and 50 yrs with a median of 5 yrs.

### 3.2. Disease Characteristics

**Figure 1:**
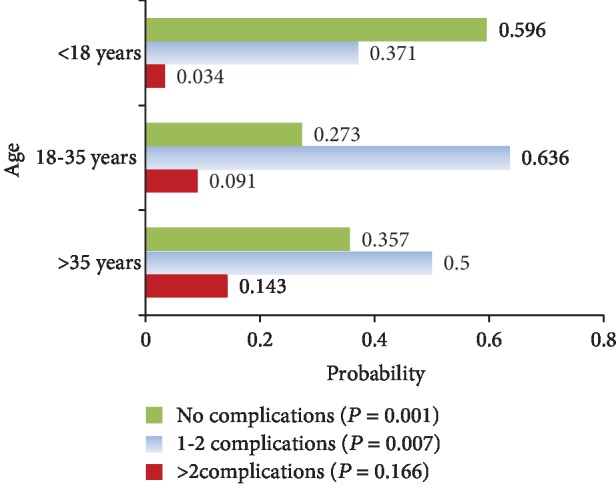
Probability of developing disease-related morbidities at different age intervals.

Ninety-three patients (58.5%) had splenomegaly, of which 32.7% had a splenectomy, while hepatomegaly and HCV were reported in 37.7% and 11.3%, respectively. The most common disease-related complications were bone disease, facial deformity (62.3%), and osteoporosis (28.3%), and 3 of those with osteoporosis had bone fractures. Endocrinopathies were second, including growth retardation (27.8%), subclinical hypothyroidism (16.8%), cholelithiasis (13.8%), PHT (11.3%), and abnormal liver function (7.5%) (Table 1). Thrombosis, EMH, and leg ulcers were less frequent, while diabetes mellitus and heart failure were not identified. Furthermore, the probability of developing the above morbidities increased significantly with age (Figure 1).

### 3.3. Transfusion History and Chelation Therapy

The median age at onset of transfusion was 4.7 years with a range between 1 and 50 yrs. Almost half of the patients (47.2%) received occasional transfusion sessions ranging from 0 to 3 per year (during severe infection, operation, or pregnancy), while just 32.1% of the patients received regular transfusion > 3/year, whereas 20.7% were never been transfused. Sixty-three patients (39.6%) received iron-chelating drugs for at least a one-year duration. Deferasirox was used in 85.7%, and deferoxamine was used in 14.3%, while 60.3% of the *β*-TI patients did not receive any type of chelating drugs. Hydroxyurea, on the other hand, was given to 47.2% of the patients.

### 3.4. Laboratory Investigations

The mean Hb at the time of enrollment was 8.9 ± 1.4 g/dL, with a range of 4.1-13.8 g/dL. The mean MCV and MCH were 72 ± 10.6 and 24.4 ± 4, respectively. The Hb F level at the time of the first presentation ranged between 4.6 and 99.5% with a mean of 65.7 ± 34.8%, while Hb A2 ranged from 0.4 to 8.4%, with a mean of 3.2 ± 2.2. Furthermore, the serum ferritin level ranged from 27 to 9882 *μ*g/L with a mean of 853.3 ± 1192.7 (Table 2). One hundred twenty-two patients (76.7%) had a serum ferritin level < 1000 *μ*g/L, while just 23.3% had a ferritin level ≥ 1000 *μ*g/L. Elevated ALT ≥ 50 IU/L, on the other hand, was reported in 12 patients (7.5%), and 9 of them (75%) had a ferritin level ≥ 1000 *μ*g/L) with a *P* value < 0.001, while none was HCV positive. HBV infection was not reported.

Selected variables were studied in a logistic regression analysis to identify their role in the pathophysiology of disease-related complications, PHT, cholelithiasis, hypothyroidism, and osteoporosis (Table 3). Age ≥ 35 was an independent risk factor for cholelithiasis and hypothyroidism. Likewise, female sex was associated with an increased risk of cholelithiasis and osteoporosis. Whereas mean serum ferritin of ≥1000 *μ*g/L was independently associated with an increased risk of osteoporosis, iron chelation therapy was protective for a multitude of other complications (PHT, cholelithiasis, hypothyroidism, and osteoporosis). Although transfusion was associated with an increased risk of osteoporosis, it was protective for cholelithiasis and hypothyroidism. Moreover, splenectomy was protective for cholelithiasis, while it was an independent risk for hypothyroidism. Finally, hydroxyurea was associated independently with increased risk of osteoporosis, though it was protective for cholelithiasis.

### 3.5. Molecular Investigations

A total of 19 different *β*-thalassemia mutations were determined among 159 TI patients; 12 (63.2%) were identified by reverse hybridization with IVS-II-I (G>A) as the most prevalent mutation, followed by IVS-I-6 (T>C) and IVS-I-110 (G>A). Other mutations were less prevalent or sporadic as shown in Table 4. On the other hand, 7 mutations were revealed by direct sequencing: CAP +1 (A>C), IVS-I-128, +20/IVS-II.745, codon 36-37, codon 82-83, IVS-II.850, and codon 127 (Table 4).

The current work had determined 37 genotypes; the most frequent was a homozygous IVS-II-1, followed by a homozygous IVS-I-6 and IVS-II-1/IVS-I-6 (Table 5). Twenty-four families (21.1%) had inherited a homozygous or compound heterozygous *β*^+^/*β*^+^ mutation, while 20.2% had the *β*^0^/*β*^+^ genotype and 56.1% had the *β*^0^/*β*^0^ genotype. Among our patients, 52.8% were the results of a consanguineous marriage. Moreover, those who inherited homozygous mutations were significantly associated with consanguinity (45.3% of patients) with a *P* value < 0.001.

## 4. Discussion

The current study had evaluated the largest cohort of *β*-TI patients in Iraq, including 159 patients from 114 families to investigate the molecular defect of *β*-TI in an attempt to improve the available diagnostic tests and current management protocols.

The inheritance of *β*^+^ mutation (*β*^+^/*β*^+^ or *β*^0^/*β*^+^) had contributed to 40.9% of *β*-TI genotypes (41.2% of the families), a result that is lower than figures from other parts of Kurdistan, Iraq (Dohuk, 54.9%; Erbil, 60.2%) [19, 20], while it is approaching figures reported from those of Baghdad (49%) [21]. Our results are also comparable to some extent to studies from India and Iran, where the inheritance of *β*^+^ alleles was not responsible for the majority of the milder *β*-thal phenotypes [22]. *β*^0^-Thal mutation, on the other hand, amounted to 56.1% of the *β*-TI family genotype, which highly suggests the coinheritance of ameliorating factors including *α*-thalassemia and/or coinheritance of a single nucleotide polymorphism (SNP) in the three major quantitative trait loci (QTLs) for the continuous synthesis of Hb F to modify the *α* : *β* chain imbalance and to reduce ineffective hemopoiesis [23]. This has been particularly emphasized in studies from Iran, where *β*^0^ mutations are more frequent in *β*-TI patients and X*mnI* polymorphism was found to be a considerable ameliorating factor [24, 25].

The relative frequency and distribution of different mutations vary in different geographical locations, and in the current study, the three most common mutations identified were IVS II-1 (47.2%), IVS I-6 (23.3%), and IVS I-110 (5%). This high prevalence of IVS II-1, a Mediterranean *β*^0^-thal mutation, is in accordance with an earlier study performed on couples attending a Sulaymaniyah premarital screening clinic, where the IVS-II-1 mutation was the most common (25.2%), although IVS I-6 amounted to 4.1% of the *β*-thal-detected mutation [26]. The later high frequency is rather expected as this study enrolled *β*-TI patients. Furthermore, the sequence of the three common mutations in this study agreed with that of Baghdad, Central Iraq [21], whereas previous studies from other parts of Kurdistan, Iraq revealed that IVS I-6 was the most frequent *β*-thal mutation, detected at around 33% [19, 20], which probably explains their higher reported frequency of *β*^+^*β*^+^ or *β*^0^*β*^+^ genotypes. Moreover, our results are consistent with that seen in different studies from Iran [24, 25, 27], including Iranian Kurds [28]. In contrast, IVS-I-6 was the most frequent *β*-thal mutation in Turkey [29], Lebanon [11], Egypt [30], Cyprus [22], and Italy [31].

Three new *β*-thal mutations were identified for the first time in Iraq, although they have been reported by earlier studies from other parts of the world. The first was the +20 (C>T) *β*^+^ mutation in the 5′ untranslated region (5′ UTR) coinherited with another *β*^+^ mutation IVS II-745 (C>T), most probably in transposition leading to the *β*-TI phenotype; otherwise, coinheritance of +20 in the *cis* position would have resulted in a *β*-thal minor phenotype [32]. The second was CAP +1 (A>C), a silent *β*^++^ Asian-Indian mutation that interacts with IVS I-1 (*β*^0^-thal mutation) resulting in a mild clinical phenotype. Finally, a dominant-like *β*-thalassemia, Hb Houston (codon 127 A>G), was also reported for the first time in Iraq (a missense mutation at codon 127 in exon III that produces an unstable hemoglobin and thalassemia intermedia phenotype in the heterozygous state) [33]. Only 5 patients (3.1%) had inherited a single *β*-thal mutation, namely, +20, IVS II-745/wt (2 patients), codon 127/wt (one patient), and IVS II-1/wt *ααα*^anti3.7^ in another 2 patients. The later genotype had resulted in increased *α* : *β* imbalance, hemolysis, and ineffective erythropoiesis [34]. We have noticed that patients with *β*^0^/*β*^+^ and *β*^0^/*β*^0^ genotypes were diagnosed at an earlier age and transfused earlier in comparison to other reported genotypes. Also, Hb F levels were the highest while Hb A_2_ levels were the lowest in *β*^0^/*β*^0^ patients. Likewise, *β*^0^/*β*^+^ and *β*^0^/*β*^0^ patients showed a higher frequency of PHT in comparison to other genotypes (Table 6).

When we compared our results with practices in TI management outlined by one of the first landmark studies “OPTIMAL CARE study” [35], which highlighted the management approaches in several Mediterranean and Middle Eastern countries (Table 7), we found that our patients were less regularly transfused with lesser numbers of splenectomized and chelated patients, whereas hydroxyurea therapy was prominently implemented at our center. Furthermore, our patients were more regularly transfused than other thalassemia centers in Iraq (Dohuk and Basra) [19, 36], Iran [37], and Italy [38] with a much higher rate of splenectomy than recent reports from Sri Lanka (12%) [39] and Qatar (7%) [40]. Despite the lower frequency of chelation therapy used in this study, 76.6% of our patients had their ferritin value < 1000 *μ*g/L, a figure that is higher than the reported value of the OPTIMAL CARE study [35] and those from previous figures from Iraq [19, 36] (Table 7). Such a finding could be attributed to the use of hydroxyurea among a higher proportion of our patients, which had improved the *α* : *β* chain imbalance and subsequently improved the ineffective hemopoiesis [12].

*β*-TI patients has had many clinical complications reported in this study despite their independence from frequent transfusion. The pathophysiology is multifactorial due to the interaction of ineffective erythropoiesis, iron overload, and chronic tissue hypoxia (chronic hemolytic anemia) [7]. The discrepancy in the rates of disease morbidities reported in various studies (Table 7) had resulted from the difference in management approaches (limited or regular transfusion, the extent of chelation, more frequent splenectomy, or use of fetal hemoglobin modulating therapy) in thalassemia centers in Iraq and worldwide [12].

This study had reported bone abnormalities as the most prevalent morbidity. Less transfusion, ineffective erythropoiesis, and eventually bone marrow expansion were directly implicated in addition to splenectomy, as well as low fetal hemoglobin [7, 38]. Osteoporosis, a well-recognized complication in *β*-TI, was correlated in this study with iron overload, female gender, transfusion, and hydroxyurea as risk factors, while lower rates were observed in patients on iron chelation therapy, in accordance with previous studies [7, 35]. Hydroxyurea was shown to be a risk factor rather than a protective factor in our study, though the clinical benefits of hydroxyurea therapy are still to be systematically established [12].

Limited data are available on endocrinopathies in *β*-TI which are reported to be less commonly seen in TI in comparison to *β*-thalassemia major [38]. The incidence of subclinical hypothyroidism, an iron-overload-related morbidity, was in accordance with previous studies from Egypt (16.7%) [41] and from Iran (19%) [42], while it was much lower than an Italian figure of 33.3% [38]. Regarding growth retardation, 27.8% had a height > 2SDbelow the 3rd percentile for the mean age and gender, a figure that is lower than previous reports from Iraq [19, 36] and Sri Lanka [39] (Table 7). This might be attributed to younger age patients and a lower proportion of splenectomized TI patients in this study as intact spleen might be a reservoir of excess body iron in addition to its scavenging effect on iron-free fraction, including nontransferrin bound iron [43].

Splenectomy in TI had been considered a significant risk factor for many disease-related complications, in particular, thrombosis and PHT [44]. In our group, two patients (1.9%) had thrombosis, and both were splenectomized. The later low incidence may be explained by younger age and the possibility of nondocumented asymptomatic cases of thrombosis. Furthermore, chronic thromboembolism in splenectomized TI patients was linked with a high frequency of PHT [45]. This study did not reveal an increased risk of PHT in splenectomized patients. Our 18 patients were diagnosed with PHT; half were splenectomized, 8/9 patients were ≤35 years, 4 used either HU therapy or chelation therapy, while another 4 used both. All of the above parameters are proposed protective factors for the development of PHT in splenectomized patients [7]. In light of the above morbidities associated with splenectomy and despite the advantage of splenectomy in maintaining higher Hb levels, clinical practice is gradually shifting to restrict indications, growth retardation, hypersplenism with symptomatic leukopenia, and/or thrombocytopenia or symptomatic hypersplenism [7, 46].

Disease-related morbidities among our *β*-TI patients potentially increased after adulthood, a result which had been suggested by a few previous studies [7, 35]. Such finding justifies an earlier intervention to curtail substantial long-term sequelae.

The current study lacked the estimation of different genetic modifiers including concomitant *α*-thalassemia and polymorphism at QTL due to lack of financial support. In addition, ferritin measurement was used instead of liver R2 magnetic imaging to determine the iron burden and PASP was estimated through Doppler echocardiography instead of right heart catheterization to diagnose PHT which may falsely raise the rate of positive results. Finally, hypogonadism was not evaluated at the time of enrollment.

## 5. Conclusions

The current study, the largest from Iraq and Kurdistan on *β*-TI, revealed that *β*^0^-thal mutation underlies 56.1% of families with milder thalassemia phenotypes in the northeastern part of Iraq, and accordingly, it is prudent to assess genetic modifiers of disease severity in *β*^0^ homozygous or compound heterozygous. Furthermore, just over half of our *β*-TI patients suffered bone diseases as a result of limited transfusion and chelation therapy. The shift towards earlier and more regular blood transfusion with iron chelation therapy to avoid serious long-term sequelae is prudent in this aspect. Finally, the impact of age on morbidities needs to be better addressed.

## Figures and Tables

**Table 1:** Patient and disease characteristics of the current study.

Parameter	Frequency	Number of evaluated patients	Percent
Demographic data			
Age (years)			
<18	90	159	56.6
18-35	55	159	34.6
>35	14	159	8.8
Gender			
Male	90	159	56.6
Female	69	159	43.4
Splenectomized	52	159	32.7
Serum ferritin (*μ*g/L)			
<1000	122	159	76.7
>1000	37	159	23.3
Treatment			
None transfused	33	159	20.7
Occasional transfusion	75	159	47.2
Regular transfusion	51	159	32.1
Iron chelation	63	159	39.6
Hydroxyurea	75	159	47.2
Complications			
Facial deformity	99	159	62.3
^∗^Osteoporosis	17	60	28.3
^∗∗^Growth retardation	17	90	18.9
^∗∗∗^Subclinical hypothyroidism	22	131	16.8
^∗∗∗∗^Cholelithiasis	19	137	13.8
Pulmonary hypertension	18	159	11.3
Abnormal liver function	12	159	7.5
Thrombosis	2	159	1.3
EMH	1	159	0.6
Leg ulcer	1	159	0.6

^∗^Osteoporosis and ^∗∗∗^subclinical hypothyroidism were evaluated in patients ≥ 10 years old and/or symptomatic, ^∗∗^growth retardation (height > 2SD below 3rd percentile for the mean age and gender) in patients ≤ 18 years, and ^∗∗∗∗^cholelithiasis estimated in 137 patients excluding the 22 patients that underwent cholecystectomy.

**Table 2:** Laboratory investigations of 159 *β*-thalassemia intermedia patients in the current study.

Laboratory tests	Mean ± SD	Range
Hb (g/dL)	8.9 ± 1.4	4.1–13.8
PCV (L/L)	26.6 ± 4.4	10.5–42.7
MCV (fL)	72 ± 10.6	48.7–116.2
MCH (pg)	24.4 ± 4	15.8–38.2
Hb A2 (%)	3.2 ± 2.2	0.4–8.4
Hb F (%)	65.7 ± 34.8	4.6–99.5
S. ferritin (*μ*g/L)	853.3 ± 1192.7	27–9882

**Table 3:** Multivariate analysis for determinants of complication rate.

Complication/parameters	RR	95% CI	*P*
PHT			
Age ≥ 35 y	0.798	0.13-4.64	0.801
Splenectomy	0.839	0.27-2.6	0.762
Transfusion	0.670	0.06-6.81	0.735
Hydroxyurea	0.600	0.19-1.82	0.369
Iron chelation	0.160	0.03-0.65	0.011
Cholelithiasis			
Age ≥ 35 y	1.153	0.19-6.73	0.874
Female	1.693	0.53-5.34	0.369
Splenectomy	0.140	0.03-0.50	0.003
Transfusion	0.537	0.05-5.27	0.594
Hydroxyurea	0.179	0.05-0.60	0.006
Iron chelation	0.325	0.08-1.19	0.09
Hypothyroidism			
Age ≥ 35 y	1.817	0.20-16.14	0.592
Splenectomy	2.178	0.70-6.75	0.178
Transfusion	0.251	0.05-1.24	0.091
Hydroxyurea	0.645	0.23-1.79	0.401
Iron chelation	0.552	0.241-1.265	0.149
Osteoporosis			
Age ≥ 35 y	0.616	0.235-1.62	0.364
Female	4.139	0.87-19.58	0.073
Ferritin ≥ 1000 *μ*g/L	6.86	1.09-42.97	0.040
Splenectomy	0.994	0.24-4.11	0.994
Transfusion	14.352	0.86-221.12	0.063
Hydroxyurea	9.004	1.67-48.41	0.010
Iron chelation	0.443	0.09-2.08	0.302

**Table 4:** *β*-Globin gene mutations in 159 thalassemia intermedia patients in the current study.

*β*-Thalassemia mutations	Frequency of allele	Percent
Very mild *β*^++^		
(1) CAP +1	2	(0.6)
(2) -101	1	(0.3)
Mild *β*^+^		
(1) IVS I.6	74	(23.3)
(2) IVS I.128	8	(2.5)
(3) IVS II.745	3	(0.9)
Severe *β*^+^		
(1) IVS I.110	16	(5)
(2) IVS I.5	1	(0.3)
*β* ^0^		
(1) IVS II.1	150	(47.2)
(2) IVS I.1	15	(4.7)
(3) Cod 8	15	(4.7)
(4) Cod 8/9	11	(3.5)
(5) Cod 5	6	(1.9)
(6) Cod 39	3	(0.9)
(7) Cod 36/37	2	(0.6)
(8) Cod 44	2	(0.6)
(9) Cod 82-83	1	(0.3)
(10) IVS II.850	1	(0.3)
(11) Cod 15	1	(0.3)
Wild	5	(1.6)
Dominant like *β*-thalassemia Cod 127	1	(0.3)

**Table 5:** Genotypes of 159 thalassemia intermedia patients in the current study.

Genotypes	Frequency	Percent
*β* ^0^/*β*^0^		
(1) IVS II.1/IVS II.1	57	35.9
(2) IVS II.1/Cod 8/9	5	3.2
(3) IVS II.1/IVS I.1	4	2.5
(4) IVS II.1/Cod 8	4	2.5
(5) Cod 8/9/Cod 8	3	1.9
(6) Cod 8/Cod 8	2	1.3
(7) IVS I.1/Cod 8	2	1.3
(8) IVS II.1/Cod 5	2	1.3
(9) IVS II.1/Cod 36/37	2	1.3
(10) IVS II.1/Cod 39	2	1.3
(11) IVS I.1/IVS I.1	2	1.3
(12) Cod 5/Cod 5	1	0.6
(13) IVS II.1/Cod 15	1	0.6
(14) IVS II.1/Cod 82-83	1	0.6
(15) Cod 44/Cod 44	1	0.6
*β* ^+^/*β*^+^		
(1) IVS I.6/IVS I.6	30	18.9
(2) IVS I.110/IVS I.110	4	2.5
(3) IVS I.128/IVS I.128	2	1.3
(4) IVS I.110/IVS I.128	1	0.6
(5) IVS I.6/IVS II.745	1	0.6
*β* ^0^/*β*^+^		
(1) IVS II.1/IVS I.6	7	4.4
(2) IVS II.1/IVS I.110	4	2.5
(3) Cod 5/IVS I.128	2	1.3
(4) IVS I.1/IVS I.6	2	1.3
(5) Cod 8/9/IVS I.110	2	1.3
(6) IVS I.1/CAP +1	2	1.3
(7) Cod 8/IVS I.6	1	0.6
(8) IVS II.1/IVS I.5	1	0.6
(9) Cod 8/9/IVS I.6	1	0.6
(10) Cod 39/IVS I.6	1	0.6
(11) IVS II.850/IVS I.6	1	0.6
(12) Cod 8/IVS I.110	1	0.6
(13) IVS II.1/IVS I.128	1	0.6
(14) IVS I.1/-101	1	0.6
*β* ^0^/wt IVS II.1/wt (*ααα*^anti3.7^)	2	1.25
*β* ^+^/wt +20, IVS II.745/wt	2	1.25
Dominant like *β*-thalassemia Cod 127/wt	1	0.6

**Table 6:** Relations of different parameters in all genotype groups.

Parameters	*β* ^0^/*β*^0^ *N* (%)	*β* ^0^/*β*^+^ *N* (%)	*β* ^+^/*β*^+^ *N* (%)	Dominant Hb Houston/wt	*β* ^+^ */*wt *N* (%)	*β* ^0^ */*wt *N* (%)	*P* value
Age at diagnosis							
Mean ± SD	6.7 ± 5.6	5.2 ± 2.6	8.6 ± 9.7	10	20.0 ± 2.8	12.5 ± 3.5	0.019
Age at first transfusion							
Mean ± SD	5.3 ± 4.4	4.8 ± 2.9	7.6 ± 10.8	8	19.0 ± 2.8	12.5 ± 3.5	0.020
Transfusion							
No	19 (21.3)	5 (18.5)	9 (23.7)	0 (0.0)	0 (0.0)	0 (0.0)	0.900
Yes	70 (78.7)	22 (81.5)	29 (76.3)	1 (100.0)	2 (100.0)	2 (100.0)	
Iron chelation							
No	48 (53.9)	19 (70.4)	28 (73.7)	0 (0.0)	0 (0.0)	1 (50.0)	0.071
Yes	41 (46.1)	8 (29.6)	10 (26.3)	1 (100.0)	2 (100.0)	1 (50.0)	
Hb A2 %							
<3.5	52 (92.9)	14 (73.7)	3 (10.3)	0 (0.0)	0 (0.0)	0 (0.0)	<0.0001
≥3.5	4 (7.1)	5 (26.3)	26 (89.7)	1 (100.0)	1 (100.0)	2 (100.0)	
Hb F %							
<50	4 (6.9)	3 (15.8)	26 (89.7)	1 (100.0)	1 (100.0)	2 (100.0)	<0.0001
≥50	54 (93.1)	16 (84.2)	3 (10.3)	0 (0.0)	0 (0.0)	0 (0.0)	
Splenectomy							
No	55 (62.5)	21 (77.8)	27 (73.0)	0 (0.0)	0 (0.0)	2 (100.0)	0.079
Yes	33 (37.5)	6 (22.2)	10 (27.0)	1 (100.0)	2 (100.0)	0 (0.0)	
PHT							
No	77 (86.5)	23 (85.2)	37 (97.4)	0 (0.00	2 (100.0)	2 (100.0)	0.036
Yes	12 (13.5)	4 (14.8)	1 (2.6)	1 (100.0)	0 (0.0)	0 (0.0)	
Hepatomegaly							
No	51 (57.3)	18 (66.7)	27 (71.1)	1 (100.0)	1 (50.0)	1 (50.0)	0.659
Yes	38 (42.7)	9 (33.3)	11 (28.9)	0 (0.0)	1 (50.0)	1 (50.0)	
Hepatitis C							
No	77 (86.5)	25 (92.1)	35 (92.1)	0 (0.0)	2 (100.0)	2 (100.0)	0.087
Yes	12 (13.5)	2 (7.4)	3 (7.9)	1 (100.0)	0 (0.0)	0 (0.0)	
Osteoporosis							
No	21 (61.8)	10 (90.9)	10(83.3)	1 (100.0)	1 (50.0)	—	0.257
Yes	13 (38.2)	1 (9.1)	2 (16.7)	0 (0.0)	1 (50.0)	—	
Cholelithiasis							
No	78 (87.6)	25 (92.6)	32 (84.2)	1 (100.0)	2 (100.0)	2 (100.0)	0.882
Yes	11 (12.4)	2 (7.4)	6 (15.8)	0 (0.0)	0 (0.0)	0 (0.0)	
Hypothyroidism							
No	67 (87.0)	18 (72.0)	31 (86.1)	1 (100.0)	2 (100.0)	2 (100.0)	0.349
Yes	10 (13.0)	7 (28.0)	5 (13.9)	0 (0.0)	0 (0.0)	0 (0.0)	
Growth retardation							
No	31 (60.8)	17 (89.5)	16 (84.2)	—	—	1 (100.0)	0.323
Yes	20 (39.2)	2 (10.5)	3 (15.8)	—	—	0 (0.0)	
ALT							
≤50	81 (91.0)	24 (88.9)	37 (97.4)	1 (100.0)	2 (100.0)	2 (100.0)	0.779
>50	8 (9.0)	3 (11.1)	1 (2.6)	0 (0.0)	0 (0.0)	0 (0.0)	
S. ferritin							
<1000	65 (74.7)	22 (81.5)	31 (83.)	1 (100.0)	1 (50.0)	2 (100.0)	0.663
≥1000	22 (25.3)	5 (18.5)	6 (16.2)	0 (0.0)	1 (50.0)	0 (0.0)	
Facial deformity							
No	22 (24.7)	13 (48.1)	22 (57.9)	1 (100.0)	0 (0.0)	2 (100.0)	0.001
Yes	67 (75.3)	14 (51.9)	16 (42.1)	0 (0.0)	2 (100.0)	0 (0.0)	

**Table 7:** Comparison of some parameters, treatment options, and disease-related complications between the current study and some other related studies.

Parameter	Current study (*n* = 159) %	Lebanon (*n* = 73) 2000 [28]	Optimal care (*n* = 584) 2010 [34]	Iran (*n* = 153) 2011 [36]	Basra (*n* = 80) 2013 [35]	Dohuk (*n* = 74) 2014 [17]	Italy (*n* = 70) 2014 [37]	Sri Lanka (*n* = 50) 2019 [38]
Splenectomized	32.7	59	55.7	46.9	^∗^	23.0	49	12.0
Serum ferritin (*μ*g/L)								
<1000	76.7	^∗^	64.4	^∗^	55	67.6	^∗^	^∗^
>1000	23.3		35.6		45	32.4		
Treatment								
Transfusion								
Never transfused	20.8	28.8	23.8	27.5	21.2	32.4	53	4.0
Occasionally	47.2	58.9	24.5	45.5	78.8	51.4	34	42
Regularly	32.1	12.3	51.7	27		16.2	13	44
Iron chelation	39.6	^∗^	47.5	^∗^	^∗^	14.9	56	46
Hydroxyurea	47.2	^∗^	34.6	^∗^	^∗^	2.7	16	^∗^
Complications								
Facial deformity	62.3	44	^∗^	^∗^	^∗^	73	^∗^	^∗^
Osteoporosis	28.3	^∗^	22.9	53	30.0	^∗^	49	^∗^
Growth retardation (height < 3rd percentile)	27.8	^∗^	^∗^	^∗^	42.5	31.3	^∗^	26.7
Subclinical hypothyroidism	16.8	^∗^	^∗^	^∗^	^∗^	^∗^	33.3	^∗^
Cholelithiasis	13.8	^∗^	17.1	9.8	2.5	^∗^	^∗^	10.0
PHT	11.3	^∗^	11	23.5	5.0	20.4	^∗^	33.3
Abnormal liver function	7.5	^∗^	9.8	29.3		13.5	^∗^	^∗^

^∗^Not mentioned in the study.

## Data Availability

The data supporting this clinical study are from previously reported studies and datasets, which have been cited. The processed data are available as PDFs once required from the corresponding author.
