# Corrigendum to “Beta-Thalassemia Intermedia: A Single Thalassemia Center Experience from Northeastern Iraq”

**DOI:** 10.1155/2020/2453270

**Published:** 2020-06-30

**Authors:** Shaema Salih Amin, Sana Dlawar Jalal, Kosar Muhammed Ali, Ali Ibrahim Mohammed, Luqman Khalid Rasool, Tara Jamel Osman

**Affiliations:** ^1^Sulaymaniyah Directorate of Health, Hewa Oncology Hospital, Sulaymaniyah, Iraq; ^2^Department of Pathology, College of Medicine, University of Sulaymaniyah, Sulaymaniyah, Iraq; ^3^Department of Medicine, College of Medicine, University of Sulaymaniyah, Sulaymaniyah, Iraq; ^4^Thalassemia and Congenital Blood Disorders Center, Sulaymaniyah 46001, Iraq

In the article titled “Beta-Thalassemia Intermedia: A Single Thalassemia Center Experience from Northeastern Iraq” [1], there was an error in Table 1. The corrected table is shown below and is listed as Table 1.

## Figures and Tables

**Table 1:** Patient and disease characteristics of the current study.

Parameter	Frequency	Number of evaluated patients	Percent
Demographic data			
Age (years)			
<18	90	159	56.6
18-35	55	159	34.6
>35	14	159	8.8
Gender			
Male	90	159	56.6
Female	69	159	43.4
Splenectomized	52	159	32.75
Serum ferritin (*μ*g/L)			
<1000	122	159	76.7
>1000	37	159	23.3
Treatment			
None transfused	33	159	20.7
Occasional transfusion	75	159	47.2
Regular transfusion	51	159	32.1
Iron chelation	63	159	39.6
Hydroxyurea	75	159	47.2
Complications			
Facial deformity	99	159	62.3
^∗^Osteoporosis	17	60	28.3
^∗∗^Growth retardation	25	90	27.8
^∗∗∗^Subclinical hypothyroidism	22	131	16.8
^∗∗∗∗^Cholelithiasis	19	137	13.8
Pulmonary hypertension	18	159	11.3
Abnormal liver function	12	159	7.5
Thrombosis	2	159	1.3
EMH	1	159	0.6
Leg ulcer	1	159	0.6

^∗^Osteoporosis and ^∗∗∗^subclinical hypothyroidism were evaluated in patients ≥ 10 years old and/or symptomatic, ^∗∗^growth retardation (height > 2SD below 3rd percentile for the mean age and gender) in patients ≤ 18 years, and ^∗∗∗∗^cholelithiasis estimated in 137 patient excluding the 22 patients that underwent cholecystectomy.
